# Sertoli cell tumor in patient with multiple intratesticular cysts

**DOI:** 10.1016/j.eucr.2022.102153

**Published:** 2022-07-04

**Authors:** Farzad Allameh, Amir Alinejad Khorram, Seyyed Ali Hojjati, Negin Rastian

**Affiliations:** a Laser Application in Medical Sciences Research Center, Shohada-e-Tajrish Hospital, Shahid Beheshti University of Medical Sciences, Tehran, Iran; b Urology and Nephrology Resaerch Center, Shahid Beheshti University of Medical Sciences, Tehran, Iran; c Department of Urology, Shohada-e-Tajrish Hospital, Shahid Beheshti University of Medical Sciences, Tehran, Iran; d Department of Pathology, Faculty of Medicine, Shahid Beheshti University of Medical Sciences, Tehran, Iran

**Keywords:** Cysts, Testis, Sertoli cell tumor

## Abstract

Sertoli cell tumor is a rare type of testicular tumor. In this study, a 70-year-old man was presented with a Sertoli cell tumor with non-classical findings. The patient noticed progressive swelling on the left hemiscrotum. Preoperative ultrasound findings were not consistent with the classic findings of the Sertoli cell tumor. Patients underwent radical left orchiectomy. The testis was multicystic and no clear parenchyma was seen. The pathology report confirmed the diagnosis of a Sertoli cell tumor.

## 1 Introduction

Testicular cancers account for about 1–1.5% of male cancers and 5% of urological cancers. It usually appears as a unilateral, painless and nodular testicular mass.

Testicular cancers are divided into germ cell tumors (GCTs) and non-germ cell tumors (NGCTs). Germ cell tumors (GCTs) account for about 90% and non-germ cell tumors (NGCTs) include about 10% of testicular cancers.

Intratesticular cysts are relatively rare, but their detection has increased with the advancement of imaging techniques. Treatment can include enucleation or even radical orchiectomy because of the malignancy risk. Differential diagnoses such as Rete Testis Adenoma, Adenomatoid Tumor, Leydig Cell Tumor, and Seminoma should be considered.^1^

Sertoli cell tumors (SCT) which are a subgroup of NGCTS, can be seen at any age but is more common in the range of 30–40 years.^2^^,^^3^

Histopathologically, there are three variants of this tumor: classic (Not Otherwise Specified (NOS)), large cell calcifying, and sclerosing subtypes. It is important to differentiate between these subtypes, as there is a difference in malignancy potential, prognosis, and treatment. No cases of malignancy have been reported among sclerosing SCT. The other two subgroups have the potential for malignancy.^3^

About 10% of SCTs are malignant and can cause metastasis. Distant metastasis can occur late after radical orchiectomy.

The prognosis of benign SCT is good but malignant types have a very poor prognosis and are difficult to treat.

Initial treatment is based on radical orchiectomy. The effect of radiotherapy on SCT has not been proven and chemotherapy is not standard in metastatic cases.^2^^,^^4^

## 2 Case report

The patient is a 70-year-old man with progressive swelling in the left scrotum. The left testicle was hard to touch and there wasn't any tenderness or pain. The patient had no history of undescended testis.

**Fig. 1:**
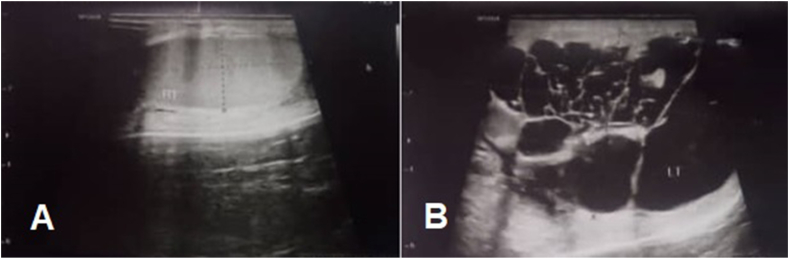
Ultrasound images of the patient. Right Testis (A) and Left Testis (B).

The ultrasound report showed a large, heterogeneous testicle with an approximate size of 77*49 mm, which contains numerous areas of cystic, septa, and calcification so that a small volume of the testicular parenchyma was visible (Fig. 1). A blood test showed a Lactate Dehydrogenase (LDH) level of 211 U/L, Alpha-Fetoprotein (AFP) 0.87 ng/ml, and Beta Human Chorionic Gonadotropins (BHCG) was <2 mlU/ml. Abdominal and pelvic computed tomography (CT) scans with oral and intravenous contrast were completely normal.

**Fig. 2:**
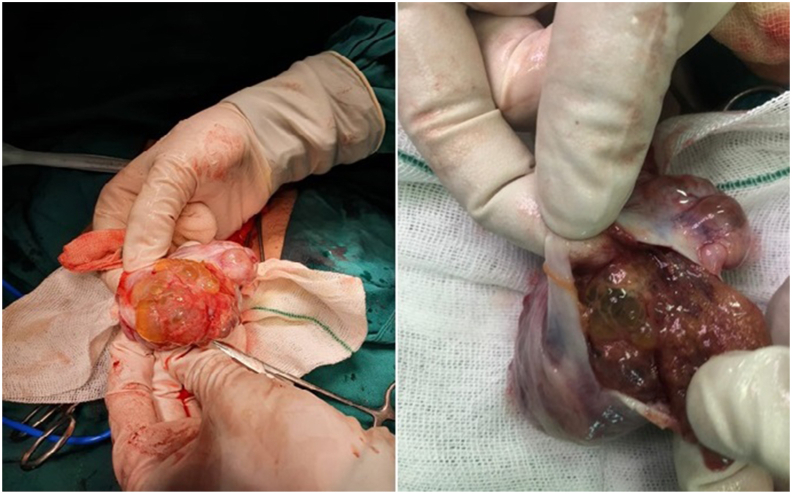
Macroscopic manifestation of the mass during surgery (multiple intratesticular cysts).

The patient underwent left radical orchiectomy. No clear testicular parenchyma was seen during surgery, and the entire tunica albuginea was filled with numerous cystic lesions (Fig. 2).

**Fig. 3:**
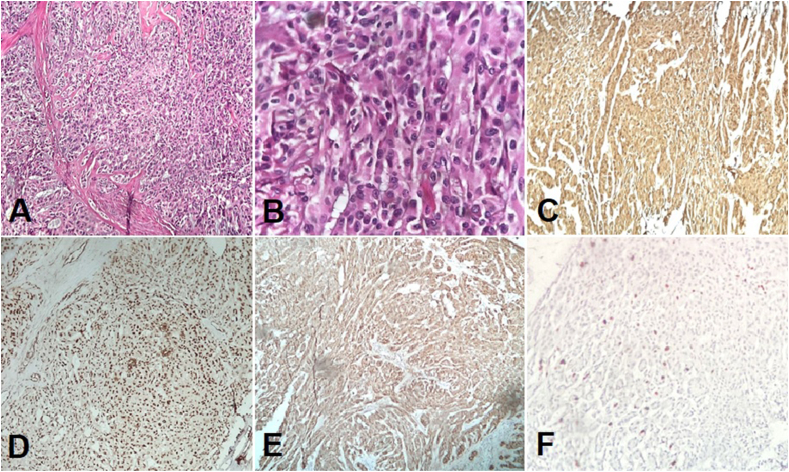
Microscopic examination of tumor showed back to back cords and few tubular structures of bland looking cells with eosinophilic cytoplasm, H&E ×100 (A) and H&E ×400 (B). Immunohistochemical examination. Calretinin (C), WT1 (D), Inhibin (E) and Ki67 index (F).

Pathologic examination revealed a testis replaced by a solid cystic mass. The solid areas' appearance was gray-yellow in color and the cystic spaces were filled with thin yellow fluid. Microscopically, the tumor was composed of a tubular and cord-like pattern lined by elongated cells. Tumoral cells had bland round to ovoid nuclei and moderated to abundant eosinophilic cytoplasm with indistinct borders separated by fibro collagenous stroma. Mitosis is rarely observed in tumors. In immunohistochemistry, the tumor cells were immunoreactive for calretinin, Wilms’ Tumor 1 (WT1), and inhibin. Ki67 index was approximately 4% (Fig. 3).

Histomorphology and immunostains were compatible with sex cord-stromal tumors in favor of the SCT, NOS subtype.

## 3 Discussion

SCT eventually accounts for about 0.4–1.5% of testicular neoplasms. It usually occurs after puberty, but can develop at any age, even in neonatal age, and is rarely seen in undescended testis.

SCT is usually sporadic but can occur in genetic syndromes such as Carney syndrome and Peutz-Jeghers syndrome.^2^

A common finding in clinical examination of these patients is painless and nontender testicular mass. Gynecomastia and hormonal disorders may be present in some patients. Premature pseudopuberty occurs in some children.^4^

There is usually a hypoechoic intratesticular lesion on ultrasound, but in our patient, there was a heterogeneous cystic lesion and a small volume of testicular parenchyma was observed.

Macroscopically, the SCT appears as a uniform or multifocal nodular mass in whitish-gray color.^4^ Manifestation of the disease as intratesticular cysts, is very rare.

Microscopically, Sertoli cells are located in tubes separated by fibrous stroma (in well-differentiated cases). One of the main criteria for histological diagnosis of SCT is the ability of these cells to form tubes.^2^ Features of malignancy in SCT include extratesticular spread, size>5 cm, high-grade cytologic atypia, >5 mitotic figures/10HPFs, necrosis, or lymphovascular invasion. About 10% of Sertoli cell tumors are malignant and are associated with metastasis. Malignant types have a very poor prognosis.^5^

Testicular cysts, especially multiple cysts, should not necessarily be considered benign. In a large study of 847 men, Hamm et al. found 34 cases of intra-testicular cysts, 16 of which were tumors.^1^ In some cases, such cysts are malignant, such as our patient whose final diagnosis was SCT. All intratesticular cysts require careful examination and should be considered important.

## 4 Conclusion

SCTs are very rare and clinical manifestations and ultrasound to diagnose them are not definitive. As in our patient, the ultrasound findings and the macroscopic appearance of the mass were not in accordance with the pathological findings. The presence of multiple cysts and the absence of normal testicular parenchyma may indicate SCT.

## Roles

**Farzad Allameh**: Conceptualization, Methodology, Visualization.

**Amir Alinejad Khorram**: Supervision, Validation, Writing- Original draft preparation.

**Seyyed Ali Hojjati**: Writing- Reviewing and Editing, Investigation.

**Negin Rastian**: Data curation, Software.

## Ethics

Patient informed consent was obtained to publish his information. The patient's private information remained confidential with the researchers.

## Financial support and sponsorship

None

## Declaration of competing interest

The authors report no conflicts of interest in this work.

## References

[1] Kang S.M., Hwang D.S., Lee J.W., Chon W.H., Park N.C., Park H.J. (2013 Apr 1). Multiple intratesticular cysts. The World Journal of Men's Health.

[2] Mhanna T., El Moudane A., Barki A., Mhanna T., Frasinescu C. (2019). Intratesticular Sertoli cell tumor: about A case and literature review. Glob J Reprod Med.

[3] Ghante Nagaraj S., Chalageri A., Vijayanand M., Gupta A. (2014 Apr 1). Sclerosing Sertoli cell tumor of the testis: case report and review of the literature. Iran J Pathol.

[4] Young R.H., Koelliker D.D., Scully R.E. (1998 Jun 1). Sertoli cell tumors of the testis, not otherwise specified: a clinicopathologic analysis of 60 cases. Am J Surg Pathol.

[5] Moch H., Cubilla A.L., Humphrey P.A., Reuter V.E., Ulbright T.M. (2016 Jul 1). The 2016 WHO classification of tumours of the urinary system and male genital organs—part A: renal, penile, and testicular tumours. Eur Urol.

